# Linsitinib, an IGF-1R inhibitor, attenuates disease development and progression in a model of thyroid eye disease

**DOI:** 10.3389/fendo.2023.1211473

**Published:** 2023-06-26

**Authors:** Anne Gulbins, Mareike Horstmann, Anke Daser, Ulrich Flögel, Michael Oeverhaus, Nikolaos E. Bechrakis, J. Paul Banga, Simone Keitsch, Barbara Wilker, Gerd Krause, Gary D. Hammer, Andrew G. Spencer, Ryan Zeidan, Anja Eckstein, Svenja Philipp, Gina-Eva Görtz

**Affiliations:** ^1^ Molecular Ophthalmology, Department of Ophthalmology, University Hospital Essen, University Duisburg-Essen, Essen, Germany; ^2^ Department of Oto-Rhino-Laryngology, Head and Neck Surgery, University Hospital Essen, University Duisburg-Essen, Essen, Germany; ^3^ Experimental Cardiovascular Imaging, Department of Molecular Cardiology, Heinrich-Heine-University Duesseldorf, Duesseldorf, Germany; ^4^ Department of Ophthalmology, University Hospital Essen, University Duisburg-Essen, Essen, Germany; ^5^ Department of Molecular Biology, University of Duisburg-Essen, Essen, Germany; ^6^ Department of Structural Biology, Leibniz-Forschungsinstitut für Molekulare Pharmakologie (FMP), Berlin, Germany; ^7^ Endocrine Oncology Program, University of Michigan, Ann Arbor, MI, United States; ^8^ Sling Therapeutics Inc., Ann Arbor, MI, United States

**Keywords:** Graves’ disease (GD), thyroid eye disease (TED), autoimmune disorder, linsitinib, IGF-1R, inflammation

## Abstract

**Introduction:**

Graves’ disease (GD) is an autoimmune disorder caused by autoantibodies against the thyroid stimulating hormone receptor (TSHR) leading to overstimulation of the thyroid gland. Thyroid eye disease (TED) is the most common extra thyroidal manifestation of GD. Therapeutic options to treat TED are very limited and novel treatments need to be developed. In the present study we investigated the effect of linsitinib, a dual small-molecule kinase inhibitor of the insulin-like growth factor 1 receptor (IGF-1R) and the Insulin receptor (IR) on the disease outcome of GD and TED.

**Methods:**

Linsitinib was administered orally for four weeks with therapy initiating in either the early (“active”) or the late (“chronic”) phases of the disease. In the thyroid and the orbit, autoimmune hyperthyroidism and orbitopathy were analyzed serologically (total anti-TSHR binding antibodies, stimulating anti TSHR antibodies, total T4 levels), immunohistochemically (H&E-, CD3-, TNFa- and Sirius red staining) and with immunofluorescence (F4/80 staining). An MRI was performed to quantify *in vivo* tissue remodeling inside the orbit.

**Results:**

Linsitinib prevented autoimmune hyperthyroidism in the *early* state of the disease, by reducing morphological changes indicative for hyperthyroidism and blocking T-cell infiltration, visualized by CD3 staining. In the *late* state of the disease linsitinib had its main effect in the orbit. Linsitinib reduced immune infiltration of T-cells (CD3 staining) and macrophages (F4/80 and TNFa staining) in the orbita in experimental GD suggesting an additional, direct effect of linsitinib on the autoimmune response. In addition, treatment with linsitinib normalized the amount of brown adipose tissue in both the *early* and *late* group. An *in vivo* MRI of the *late* group was performed and revealed a marked decrease of inflammation, visualized by ^19^F MR imaging, significant reduction of existing muscle edema and formation of brown adipose tissue.

**Conclusion:**

Here, we demonstrate that linsitinib effectively prevents development and progression of thyroid eye disease in an experimental murine model for Graves’ disease. Linsitinib improved the total disease outcome, indicating the clinical significance of the findings and providing a path to therapeutic intervention of Graves’ Disease. Our data support the use of linsitinib as a novel treatment for thyroid eye disease.

## 1 Introduction

Thyroid eye disease (TED) is the most common extra thyroidal manifestation of the autoimmune condition Graves’ disease (GD), which is caused by autoantibodies against the thyroid stimulating hormone receptor (TSHR) leading to overstimulation of the thyroid gland (1, 2). The action of autoreactive T-cells and binding of autoantibodies to antigens within the orbital tissue results in activation of orbital fibroblasts (OFs), differentiation into adipocytes and myofibroblasts and hyaluronan production (3). The insulin-like growth factor 1 receptor (IGF-1R) is another surface protein that is overexpressed by orbital fibroblasts as well as by B- and T-cells in TED and GD patients (4–6). TSHR and IGF-1R together form complexes via crosstalk (7, 8), which thus in concert with each other aggravate and mediate the disease finally leading to orbital inflammation, proptosis and squinting in TED patients (8–10).

In this study, we evaluate the effect of linsitinib, also known as OSI-906, a novel, highly selective small-molecule dual inhibitor of the IGF-1R and IR (11) on the outcome of Graves’ disease. The IGF-1R exists as a homodimer, where each protomer consists of an extracellular ectodomain, a transmembrane and a cytoplasmic tyrosine kinase domain (12). This tyrosine kinase domain mediates the intrinsic tyrosine kinase activity, which is stimulated upon binding of the corresponding ligands, IGF-I and IGF-II. The intrinsic activation of the receptor then results in autophosphorylation and stimulation of several signaling cascades (13). Linsitinib effectively inhibits the intrinsic tyrosine kinase activity of the IGF-1R by binding to the cytoplasmic tyrosine kinase domain (13). Specifically, linsitinib inhibits the autophosphorylation after binding of the cognate ligands to the IGF-1R and IR, and therefore blocks the IGF-I and IGF-II induced activation of downstream pathways, such as AKT and ERK signaling (13).

Linsitinib, an investigational drug administered via oral tablet, has been evaluated in several oncology clinical trials encompassing more than 800 patients (11, 14–17). Recently, the FDA authorized initiation of a clinical study which aims to evaluate the safety and efficacy of linsitinib in patients with active TED (NCT05276063).

In the present study, we investigated the efficacy of linistinib for active and chronic TED in our experimental mouse model. The mouse model is based on multiple immunizations with a plasmid encoding the A-subunit of the human TSHR followed by electroporation (18, 19).

We demonstrate a marked beneficial effect of the drug on the outcome of experimental TED. These results are consistent with the therapeutic hypothesis being evaluated in an ongoing Phase 2b clinical study, and provide essential context for the assessment of linsitinib’s potential to become an additional first line treatment option for TED patients.

## 2 Methods

### 2.1 Mice

Female BALB/c inbred mice age 6 weeks were purchased from Envigo Netherlands GmbH and accommodated under specific conditions described before (20). All animal procedures were reviewed and approved by the North Rhine Westphalian State Agency for Nature, Environment and Consumer Protection (LANUV), Germany. In particular, all experiments were performed according to the FELASA regulations and we also followed the ARRIVE guidelines.

### 2.2 Immunizations

**Figure 1:**
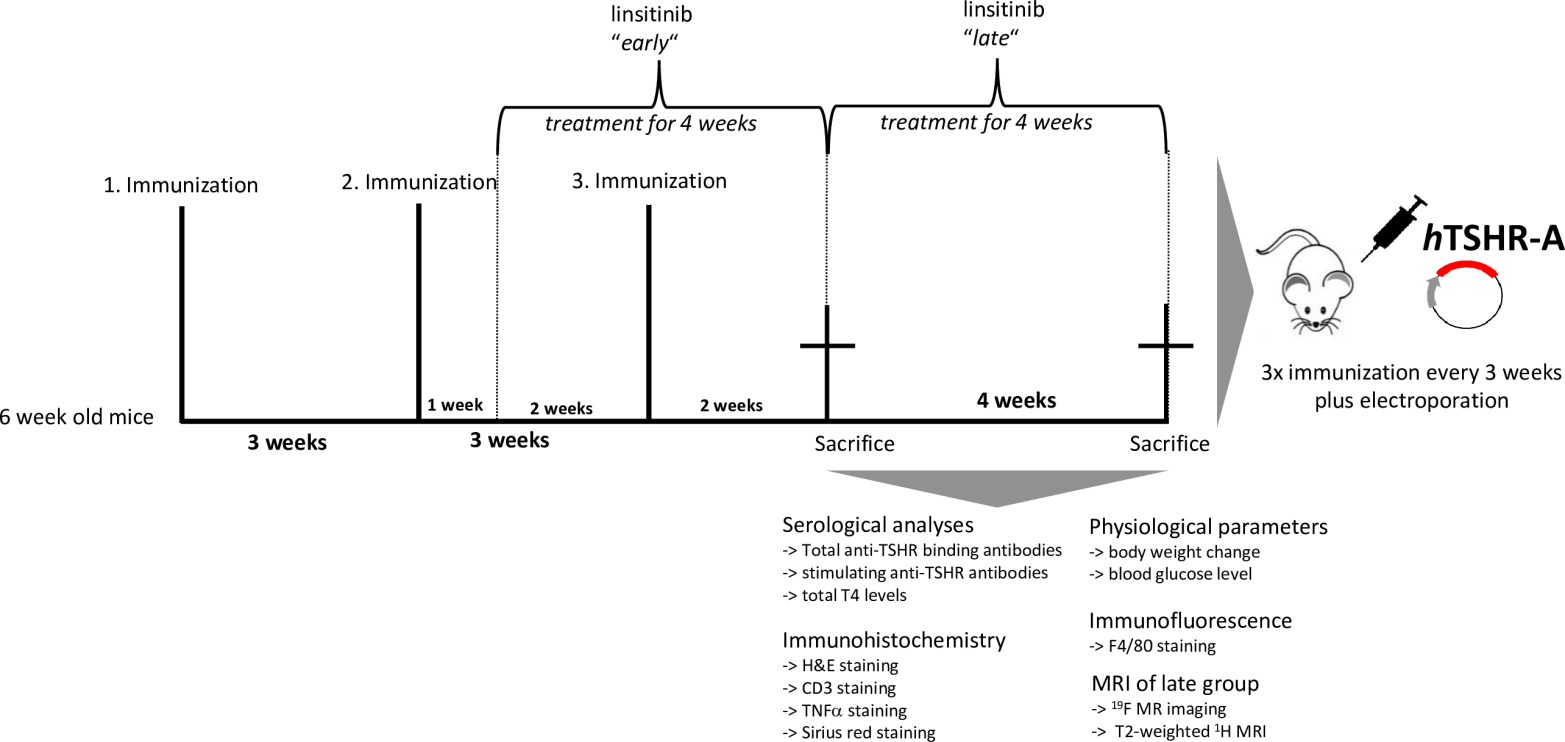
Experimental design of linsitinib treatment in a mouse model for Graves’ disease. In order to induce autoimmune hyperthyroidism and associated thyroid eye disease, female BALB/c mice at age of six weeks were immunized with a TSHR A-subunit encoding plasmid three times with three weeks intervals apart. THSR-immunized mice were either treated with linsitinib one week after the second immunization for four weeks (linsitinib “*early*”) to evaluate the effect of linsitinib in the active state of the disease, or treated with linsitinib two weeks after the second immunization for four weeks to evaluate the effect in a more chronic state of the disease (linsitinib “*late*”). Two groups of TSHR-immunized mice remained untreated. Female BALB/c mice immunized with a ß-Gal encoding control plasmid and served as healthy control mice (ß-Gal mice). The experiment was terminated six weeks after the last immunization and several investigations, which are also displayed in the figure, were performed on the different mouse groups.

Five groups of 10 animals/group were immunized with either the eukaryotic expression plasmid pTriEx1.1Neo-human (h)TSHR A-subunit (also known as hTSHR289) or the control pTri1Ex1.1Neo-ß-Gal plasmid (control ß-Gal group) as previously described (18, 20). Briefly, the mice were sedated with isoflurane and 50 µg (1mg/ml) of plasmid was injected into each M. biceps femoris, followed by electroporation. The electroporation process was performed using the BTX Gemini x2 wave electroporator with a 7-mm stainless steel (SS)- electrode disc tip by Harvard Apparatus. Application of the current was performed ten-20 millisecond wave pulses to gradually permeate the cell membranes. This ensures the transfer of the plasmid, while on the other hand also avoiding additional damage to the muscle tissue (21). Mice were randomly allocated to the groups by a technician not involved in the study. All mice underwent three immunizations three weeks apart. The first two TSHR groups, in which one group received *early* linsitinib treatment, were sacrificed two weeks after the last immunization. The other two TSHR groups, in which one group received *late* linsitinib treatment were sacrificed six weeks after the last immunization. The control β-Gal group was sacrificed six weeks after the last immunization as well (**Figure 1**).

### 2.3 Linsitinib treatment

Linsitinib was administered daily, 7 days/week, via oral gavage. The dosing volume was 150μl and the dosing vehicle was 25 mM tartaric acid. Ready-to-use solution was made freshly every five days and stored at room temperature while protected from light. The investigation of linsitinib efficacy was divided into two treatment strategies: *early* and *late* treatment (two TSHR immunized groups each). For *early* therapy, one TSHR group received linsitinib for four weeks starting one week after the second immunization. Another TSHR group received the vehicle, tartaric acid, for the same time. After the end of the treatment, the mice were sacrificed.

For *late* therapy, linsitinib was administered to one TSHR group for four weeks starting two weeks after the third immunization. Another TSHR group and the ß-Gal control group received the vehicle for the same time. After the end of the treatment the *late* mouse group was examined via MRI and sacrificed.

### 2.4 Linsitinib treatment of early group

Treatment with linsitinib started one week after the second immunization for four weeks.

Prior to this study, linsitinib had never been evaluated in BALB/c mice immunized with human TSHR-A, so in the beginning all nine mice of the early group underwent a dose adapting process. An initial dose of 60 mg/kg was not well-tolerated and resulted in overall decreased activity and weight loss. Reducing the dose to 30 mg/kg parallel to the last (third) immunization continued to be associated with decreased activity and weight loss. Further dose exploration in the range of 5-15 mg/kg identified 10 mg/kg as the best dose to ensure tolerability through the duration of the study. This dosing study allowed us to define an effective and tolerable dose for treatment in this BALB/c mouse model of Graves’ disease. All mice received a cumulative dose of 280 mg/kg in average at the end of the 4 weeks of treatment.

### 2.5 Linsitinib treatment of late group

Linsitinib was administered daily starting two weeks after the third immunization for four weeks with a dose of 10 mg/kg in 150 μl vehicle (25 mM tartaric acid in water) via oral gavage. The mice showed no severe objective adverse effects and received a cumulative dose of 280 mg/kg during the 4 weeks of treatment.

### 2.6 Magnetic resonance imaging

MRI was performed right before the sacrifice of the *late* group and 8 mice per group were randomly selected for measurement. Animals were injected in the tail vein with 300 µl [^19^F] Perfluorocarbon Nanoemulsion (PFC) for *in situ* labeling of macrophages/monocytes and subsequent tracking by [^19^F] MRI. Measurements were performed as described previously (22, 23). Mice were anaesthetized with 1.5% isoflurane during the whole procedure that took between 60 and 90 minutes. Body temperature and vital functions were monitored the whole time.

### 2.7 Serological analyses

Total anti-TSHR binding antibodies, stimulating anti-TSHR antibodies and total T4 levels were analyzed in sera as previously described (23). Briefly, to determine total anti-TSHR binding antibody titers, 25 μl serum was combined with 75 μl human control serum and the antibody concentration was measured using a commercial TSH receptor antibody concentration (TRAK) kit following manufacturer´s instructions (Thermo Fisher Scientific Cat# 701.1, RRID : AB_2927546.). CHO cells stably transfected with mouse TSHR, kindly provided by Sandra McLachlan, were used to perform a bioassay in order to measure stimulating anti-TSHR antibodies. cAMP production of the cells was measured by ELISA (Enzo Life Sciences Cat# ADI-900-067, RRID : AB_2814712). Total T4 concentrations were determined via ELISA following the instructions of the vendor (DRG International Cat# EIA-1781, RRID : AB_2927536).

### 2.8 Histopathology and immunohistochemistry of thyroids and orbits

Formalin fixed and paraffin embedded thyroids samples were sectioned at 1 μm, deparaffinized and H&E stained. Middle sections were used for histological analyses. The morphology was analyzed by an investigator blinded to the identity of the samples. The samples were indexed as normal, heterogeneous or hyperthyroid in comparison to healthy control mouse thyroids. Thyroid tissue was stained for CD3 employing a rabbit polyclonal anti-CD3-IgG antibody (dilution 1:25; Agilent Cat# A0452, RRID : AB_2335677) and positive cells were counted. Images were generated on the Olympus BX51 microscope. Orbital sections were also embedded in paraffin, deparaffinized, H&E stained and areas of nerve, fat and muscle tissue were quantified using ImageJ software. Fat composition was determined by measuring areas of white and brown fat. Orbital tissue was also stained for CD3 as above. Macrophages were stained employing the primary monoclonal rat, anti-mouse, anti-F4/80 antibody (dilution 1:250; Abcam Cat# ab6640, RRID : AB_1140040) and the secondary Cy3 donkey, anti-rat F(ab’)_2_ fragment IgG (dilution 1:100; Jackson ImmunoResearch Labs Cat# 712-166-153, RRID : AB_2340669). Positive cells were imaged using the Leica TCS SL confocal fluorescence microscope and counted in orbital fat and muscle tissue.

For TNFα, a rabbit polyclonal anti-TNFα-IgG antibody was used (dilution 1:200, (Elabscience Cat# E-AB-33121, RRID : AB_2923019) and visualized using an HRP-conjugated-Polymer-System following exactly the manufacturer´s manual (Zytomed Systems). Positive cells were imaged using Olympus BX51 upright microscope and counted in orbital fat and muscle tissue. For representation of the collagen fibers section were stained with Sirius red and the intensity was determined with ImageJ software.

### 2.9 Statistics

Statistical analyses including the Z-score method were performed using GraphPad Prism Software (Prism 7, RRID : SCR_002798) and accomplished with One‐Way‐ANOVA for multiple comparisons between the different mouse groups and with an unpaired t-test for the comparison between two different mouse groups. Data is presented with ± standard deviation (SD). P-values are marked as follows: *p ≤ 0.05; **p ≤ 0.01; ***p ≤ 0.001; ****p ≤ 0.0001. Changes between mouse groups with p‐values > 0.05 are regarded as not statistically significant and are not shown in the graphs. Additionally, the upper 99% confidence interval (CI) of the healthy control β‐galactosidase group was defined as threshold for positivity of individual mice and is indicated by a dotted line when appropriate. The standard score (Z‐score) was used to compare the results from the different mouse groups normalized to the mean value of the total mouse population (reference population). The Z‐score values are given in arbitrary units and represent the values of standard deviation from the mean value of the reference population.

## 3 Results

### 3.1 Linsitinib does not alter formation of anti-TSHR autoantibodies in Graves’ disease

**Figure 2:**
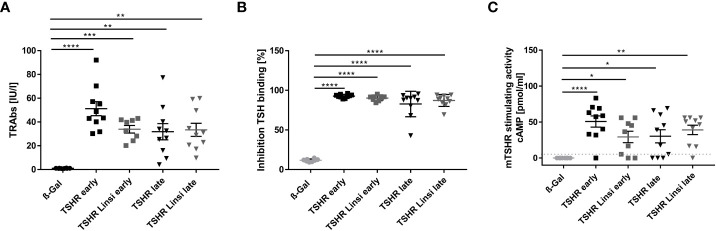
Effect of linsitinib treatment on formation of autoantibodies against TSHR. **(A, B)** Total anti-TSHR binding antibody titers (TRAbs) in serum samples obtained from mice from each group, i.e., ß-Gal, TSHR *early*, TSHR *early* treated with linsitinib (TSHR Linsi *early*), TSHR *late*, TSHR *late* treated with linsitinib (TSHR Linsi *late*) **(A)** and inhibition of TSH binding to the TSHR by the autoantibodies **(B)** were determined by an ELISA. **(C)** Stimulating activity of anti-mTSHR antibodies (TSAbs) was evaluated by measuring cAMP production in CHO cells treated with autoantibodies. Shown are the mean ± SD, n=8 for ß-Gal, n=10 for TSHR *early*, TSHR *late* and TSHR Linsi *late* and n=9 for TSHR Linsi *early*. Statistical differences were determined using one-way-ANOVA; *p < 0.05, **p < 0.001, ***p < 0.001, ****p < 0.0001. The upper 99% CI of the TSHR-stimulating activity in the ß-Gal group is indicated by a dotted line.

To evaluate the efficacy of linsitinib on the formation of autoantibodies the total anti-TSHR binding antibody (TRAb) titer was measured. Immunization of mice with the TSHR A-subunit induced high anti-TSHR binding antibody titers in the sera (**Figure 2A**). The healthy ß-Gal immunized control mice did not produce anti-TSHR binding antibodies (**Figure 2A**). In the *early* state of the disease immunized mice produced higher titers of anti-TSHR binding antibodies than in the *late* state. This indicates a more active and severe autoimmune response in the *early* state, which seems to flatten during the course of the disease (**Figure 2A**). Linsitinib had no effect on the formation of anti-TSHR binding antibodies in the *late* state of the disease, whereas in the *early* state of the disease a trend towards lower levels of anti-TSHR binding antibodies was seen, although this trend was not significant (**Figure 2A**). Antibodies induced by immunization blocked TSH binding, indicating the functional significance and relevance of these antibodies (**Figure 2B**). Linsitinib did not alter inhibition of TSH binding properties of the antibodies (**Figure 2B**). This is consistent with the fact that linsitinib does not bind to TSHR. Next, we determined whether anti-TSHR stimulating antibodies induce cAMP formation in target cells. Anti-TSHR stimulating antibodies mimic the action of TSH, which binds to the TSHR, followed by an activation of signaling cascades with a consecutive release of cAMP (24). Stimulating anti-TSHR antibodies are specific biomarkers for GD and TED and are responsible for many of its clinical manifestations (24). Our studies revealed that 9/10 animals in the *early* TSHR-immunized mouse group and 6/10 in the *late* TSHR-immunized mouse group produced antibodies after immunization that stimulated the endogenous mouse TSHR (mTSHR) receptor (**Figure 2C**). Linsitinib treatment had no effect on the formation of stimulating anti-mTSHR antibodies in the *late* treated group, but was associated with a modest, but not significant trend towards lower levels in the *early* treated group. Only 6/9 animals developed stimulating antibodies after treatment with linsitinib (**Figure 2C**) in comparison to 9/10 animals in the untreated immunized group.

### 3.2 Linsitinib effectively reduces autoimmune hyperthyroidism in the early state of the disease

**Figure 3:**
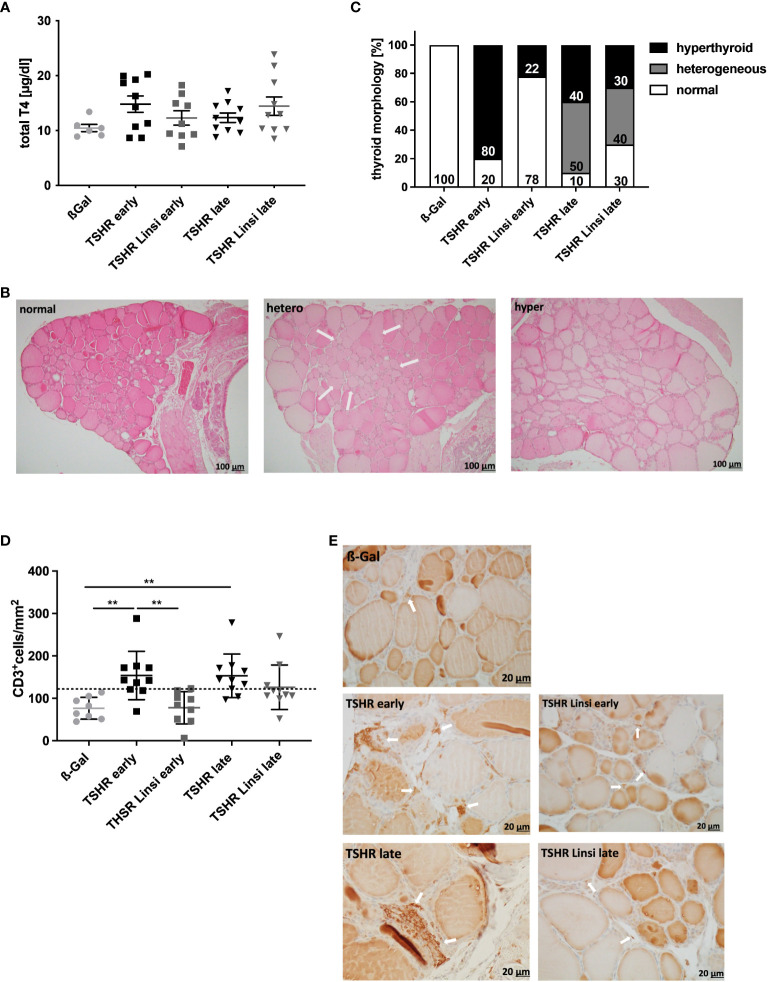
Linsitinib treatment improves thyroid dysfunction. **(A)** Serum T4 values were measured by an ELISA. **(B)** The thyroid glands of all mice of each group were fixed, paraffin embedded and sections (1µm) of the middle thyroidal area were H&E stained. Representative images of normal, heterogeneous or hyperplastic thyroid morphology are shown (magnification x10). Arrows indicate hyperplastic parts within normal morphology. **(C)** Thyroid morphology was classified as normal, heterogeneous or hyperplastic and is given in % of each group. **(D)** Sections of the middle thyroidal area were immune histologically stained for CD3 and positive T-cells were counted. **(E)** Representative images of CD3 staining for each mouse group are shown (magnification x40). Arrows indicate CD3+ T-cells in the thyroid tissue. Given are the mean ± SD **(A,D)** and representative stainings **(B, E)**, n=8 for ß-Gal, n=10 for TSHR *early*, TSHR *late* and TSHR Linsi *late* and n=9 for TSHR Linsi *early*. Statistical differences were determined using one-way-ANOVA; **p < 0.001. The upper 99% of the CD+ T-cell numbers and fT4 in the ß-Gal group is each indicated by a dotted line.

Next, we investigated the effect of linsitinib treatment on hyperthyroidism by measuring thyroid hormone T4 values and by evaluating thyroid histopathology (**Figure 3**). The total T4 values of all TSHR immunized groups were not significantly different compared to total T4 values of the healthy control ß-Gal immunized group (**Figure 3A**). The total T4 levels were elevated in 6/10 of the *early* TSHR- control mice compared to the upper 99% of the ß-Gal group and in 4/10 of the *late* TSHR- control mice (**Figure 3A**). Linsitinib had no effect on total T4 levels.

Thyroid sections were stained with H&E to identify morphological changes indicating an autoimmune hyperthyroidism (**Figure 3B, C**). Thyroids were categorized into normal, heterogeneous, and hyperplastic morphology. Hyperplastic morphology was characterized by cylindrical follicular cells with thick, multilayer epithelium, faded colloid, dividing follicles and asymmetrical thyroid lobes, findings that in summary indicate follicular hyperactivity. Some of the glands showed heterogeneous morphology with a mixture of normal appearing zones and areas of hyperplastic follicles (**Figure 3B**). Characteristic changes of the morphology, indicative for hyperthyroidism were found in 80% of *early* TSHR-immunized mice but only in 22% of immunized mice after linsitinib treatment (**Figure 3C**). Linsitinib, therefore, prevented morphological changes of the thyroid indicative of hyperthyroidism. In the *late* TSHR-immunized group, hyperthyroidism was found in 40% of individuals and a heterogeneous image was detected in 50% of the mice. Linsitinib treatment reduced hyperthyroidism in the *late* THSR-immunized group to 30% of all mice and the appearance of heterogenous thyroid glands to 40% (**Figure 3C**).

Immunehistological staining of thyroid sections revealed extensive T‐cell infiltration into the interfollicular connective tissue in TSHR‐immunized mice. Specifically, 70% of the *early* and *late* TSHR-immunized mice showed increased T-cell infiltration in comparison to healthy ß-Gal control mice (**Figure 3D**). Linsitinib prevented infiltration of thyroid tissue with CD3+ T-cells in *early* TSHR-immunized mice, showing no significant differences compared to the healthy ß-Gal immunized control group (**Figure 3D**). In the *late* TSHR-immunized mice linsitinib was without significant effect. However, linsitinib-treatment reduced the number of mice with increased T-cell infiltration in the *late* group in comparison to healthy ß-Gal control mice from 70% in the untreated immunized group to 30% (**Figure 3D**), although the variation is too high to reach statistical significance.

### 3.3 Linsitinib slightly decreases body weight and does not modify blood glucose levels

**Figure 4:**
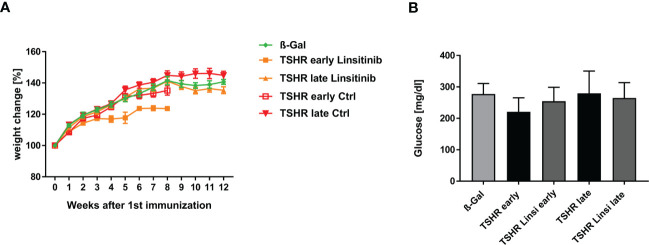
Physiological *in vivo* parameters in response to linsitinib treatment. **(A)** The weights of all mice were measured daily during the study. **(B)** Glucose was measured in blood using a blood glucose meter at the end of the experimental period. **(A)** Statistical differences were determined using the area under the curve (AUC), followed by analysis with a student t-test. **(B)** Shown are the mean ± SD, n=8 for ß-Gal, n=10 for TSHR *early*, TSHR *late* and TSHR Linsi *late* and n=9 for TSHR Linsi *early*. Statistical differences were determined using one-way-ANOVA.

The body weight of all mice was determined daily during the experimental period. Linsitinib treatment reduced the body weight of the *early* TSHR group, as seen in **Figure 4A**, however not statistically significant.

To further analyze the weight change of the mice, we determined the area under the curve (AUC). For the TSHR *early* group and the TSHR Linsi *early* group the corresponding AUC were determinate and compared via t-test to each other and to the AUC of the ß-Gal group until week 8, due to the sacrifice of the *early* group after week 8. An unpaired t-test of the ß-Gal group vs. TSHR *early* (p= 0.6912), ß-Gal vs. TSHR Linsi *early* (p= 0.9626) and TSHR *early* vs. TSHR Linsi *early* (0. 2175) revealed no statistically significant differences. These results demonstrate that the weight decrease of the TSHR Linsi *early* group stabilized after the dose adapting period. The AUC of the TSHR *late* group and the TSHR Linsi *late* group was compared via t-test to each other and to the AUC of the ß-Gal group (for the whole 12 weeks). An unpaired t-test of the ß-Gal group vs. TSHR *late* (p= 0.5507), ß-Gal vs. TSHR Linsi *late* (p= 0.9962) and TSHR *late* vs. TSHR Linsi *late* (0. 4218) revealed no statistically significant differences.

To determine, whether linsitinib has an effect on the blood glucose level, as the IGF-1R and the IR play a pivotal role in mediating the glucose metabolism (13), and thus inhibiting those receptors can potentially interfere with the glucose homeostasis, blood glucose levels were measured. Although linsitinib is known at higher doses to impact blood glucose levels in mice (13) we did not observe an effect of linsitinib on blood glucose when administered daily at 10 mg/kg over four weeks (**Figure 4B**).

### 3.4 Thyroid eye disease is improved by linsitinib treatment

**Figure 5:**
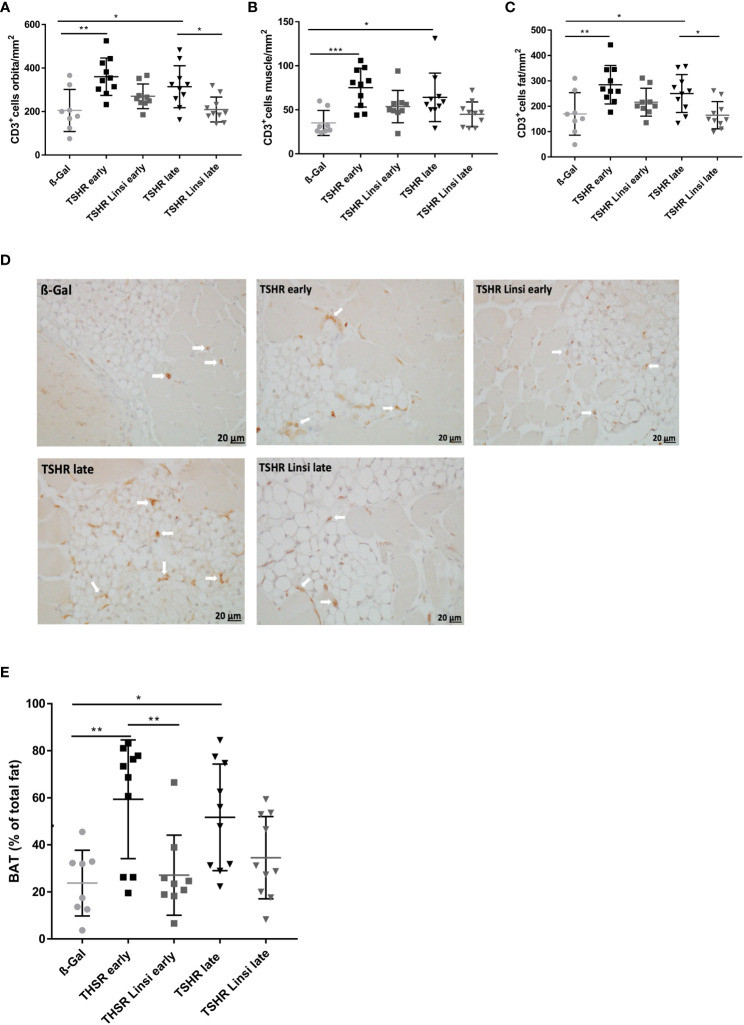
Orbital T-cell infiltration and tissue remodeling improved upon linsitinib treatment. Orbitae were fixed, paraffin embedded and consecutive sections of the middle orbital area were stained for CD3+ cells. **(A–C)** The number of CD3+ T-cells was counted in the total orbita sections **(A)**, in the orbital fat **(B)** and in the orbital muscle **(C)**. **(D)** Representative images of the total orbita stained for CD3 (magnification x40) are shown in panel. Arrows indicate CD3+ T-cells within the orbital tissue. **(E)** The area of brown adipose tissue was determined in the sections and is given as percentage of the total area of fat. Displayed are the mean ± SD **(A–C, E)** and representative stainings **(D)**, n=8 for ß-Gal, n=10 for TSHR *early*, TSHR *late* and TSHR Linsi *late* and n=9 for TSHR Linsi *early*. Statistical differences were determinedusing one-way-ANOVA; *p < 0.05,**p < 0.001, ***p < 0.001 .

**Figure 6:**
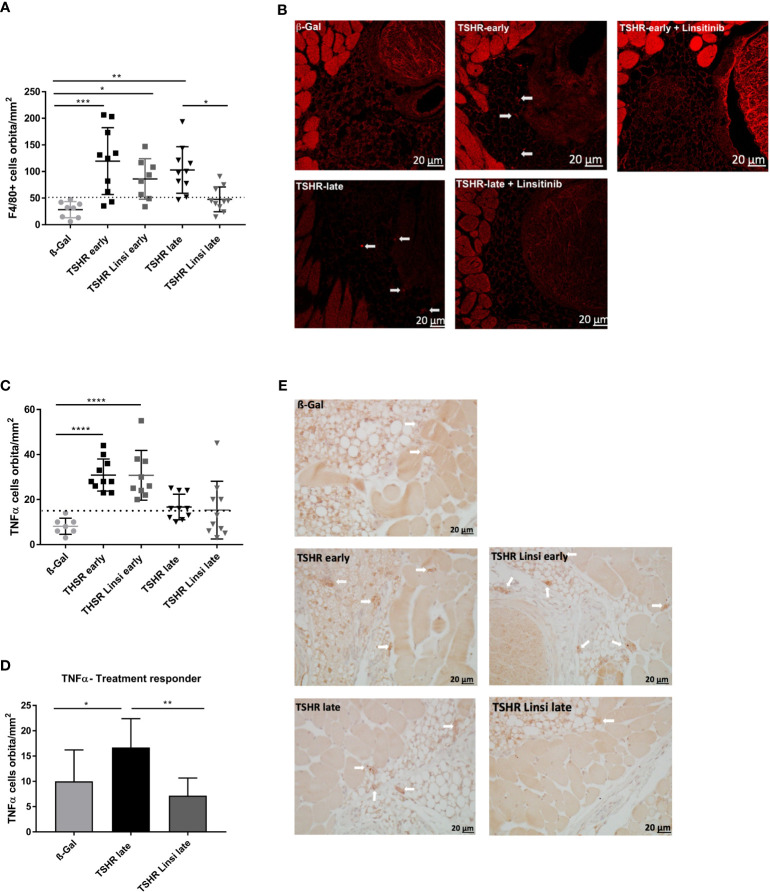
Effect of linsitinib on orbital macrophage infiltration. Orbitae were removed, fixed, paraffin embedded and serial sections of the middle orbital area were stained with anti-F4/80 antibodies as a marker for macrophages. **(A)** Representative images of immunostainings for F4/80 are shown (magnification x40) **(B)** Arrows indicate F4/80 positive macrophages within the orbital tissue in *early* and *late* TSHR immunized mice. The macrophages can be found in different microscopic levels, which requires to focus through the tissue while counting the positive cells. For this reason, the background of the TSHR-*late* image appears darker. **(B)** Orbitae were removed, fixed, paraffin embedded and serial sections of the middle orbital area were stained for TNFα and positive cells in the orbit were counted. **(C)** The mice in the *late* group were divided in responder and non-responder. i.e., those under the dotted line, within the area of the ß-Gal mice, and the number of TNFα positive macrophages in the orbit was again determined. **(D)** Representative images of immunostainings for TNFα are shown (magnification x40). **(E)** Arrows indicate TNFα+-positive macrophages within the orbital tissue. Presented are the mean ± SD **(A, C, D)** and representative stainings **(B, E)**, n=8 for ß-Gal, n=10 for TSHR *early*, TSHR *late* and TSHR Linsi *late* and n=9 for TSHR Linsi *early*. Statistical differences were determined using one-way-ANOVA; *p < 0.05, **p < 0.001, ***p < 0.001, ****p < 0.0001. The upper 99% CI of the number of macrophages in the ß-Gal group is indicated by a dotted line.

To evaluate whether and to which degree linsitinib reduces thyroid eye disease, consecutive sections of the orbita were stained for CD3+ T-cells, brown adipose tissue and macrophages with a F4/80 and TNFα staining. (**Figures 5**, **6**). Immunostaining for CD3+ T-cells revealed a severe T-cell infiltration into the orbital fat and muscles in TSHR-immunized mice in the *early* and *late* group compared to ß-Gal mice (**Figure 5A**). Treatment with linsitinib significantly improved T-cell infiltration in the *late* TSHR-mice (**Figure 5A**) and also reduced T-cell numbers in the orbita of the *early* group (p=0,1), which however did not statistically differ due to the relatively high variation.

To evaluate, whether T-cell infiltration in the orbital fat and the orbital muscles differs between treated and untreated groups, the graphs were divided into CD3+ T-cells in orbital fat (**Figure 5B**) and in orbital muscles (**Figure 5C**). T-cell infiltration was more severe in orbital fat than in orbital muscles in both, the *early* and *late* TSHR-group (**Figures 5B, C**). Linsitinib significantly reduced T-cell infiltration in the orbital fat of the *late* TSHR-immunized mice (**Figure 5B**), while the *early* group showed a trend to reduced T-cell infiltration after treatment with linsitinib (p= 0.19). A representative staining of the orbita for T-cells is shown in **Figure 5D**.

An increased percentage of brown adipose tissue was found in the orbital tissue of *early* and *late* TSHR immunized control mice compared to healthy control ß‐Gal mice (**Figure 5E**). Treatment with linsitinib normalized the amount of brown adipose tissue in both, the *early* and *late* TSHR-immunized group, showing no significant differences compared to the ß-Gal group anymore (**Figure 5E**). In the *early* state of the disease the effect of the drug was higher than in the *late* group (**Figure 5E**). In the *late* TSHR-immunized group the effect of linsitinib was statistically not significant, but a trend towards lower brown adipose tissue levels was detected (p=0,3) (**Figure 5E**).

Macrophage infiltration was evaluated by immunofluorescence staining with F4/80 (**Figures 6A, B**) and immunostaining for TNFα (**Figures 6C-E**). F4/80 is a cell surface molecule which is highly expressed on murine monocytes and tissue macrophages (25), whereas TNFα is a proinflammatory cytokine which is known to be highly produced by macrophages during inflammation, injury or infection and thus mainly indicates pro-inflammatory M1 macrophages (26). Infiltration of macrophages was more severe in the *early* state of the disease than in the *late* state, seen in both the F4/80 staining (**Figure 6A**) and the TNFα staining (**Figure 6C**). Linsitinib had no effect on macrophage infiltration in the *early* TSHR-immunized group (**Figures 6A, C**). However, treatment with linsitinib significantly reduced F4/80 indicated macrophage infiltration in the *late* TSHR-immunized group (**Figure 6A**). Regarding TNFα, no significant macrophage infiltration was detected in the *late* TSHR-immunized group (**Figure 6C**). However, closer inspection of the *late* TSHR-immunized group treated with linsitinib reveals a group responding to linsitinib treatment and a non-responder group (**Figure 6D**). The responder group consists of the 6 mice below the dotted line, indicating the upper 99% CI of the macrophage numbers in the ß-Gal mice orbita. Comparing the *late* TSHR-immunized group with the *late* group responding to linsitinib reveals a significant reduction of TNFα secreting macrophages in the orbita after treatment with linsitinib (**Figure 6D**).

### 3.5 Linsitinib does not alter fibrosis in Graves’ disease

**Figure 7:**
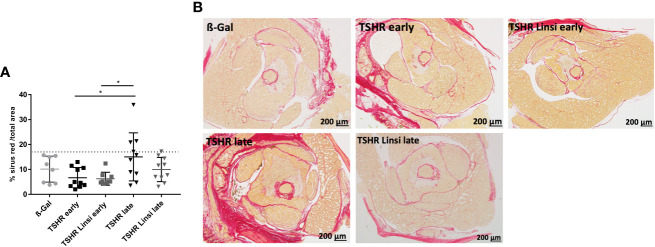
Effect of linsitinib on orbital fibrosis. Orbitae were removed, fixed, sectioned and stained with Sirius red. **(A)** The percentage of fibrosis was evaluated in the orbital sections using polarization filter and ImageJ after staining with Sirius red. **(B)** Representative images of staining for Sirius red are shown (magnification x4). Presented are the mean ± SD **(A)** and representative stainings **(B)**, n=8 for ß-Gal, n=10 for TSHR *early*, TSHR *late* and TSHR Linsi *late* and n=9 for TSHR Linsi *early*. Statistical differences were determined using one-way-ANOVA; *p < 0.05. The upper 99% of Sirius red intensity in the ß-Gal group is indicated by a dotted line.

Next, we analyzed the effect of linsitinib on orbital fibrosis by staining with Sirius red. However, the TSHR-immunized mice did not significantly develop an orbital fibrosis compared to the healthy ß-Gal group (**Figures 7A, B**). In the *late* TSHR-immunized group 3/10 mice showed a trend towards fibrosis, whereas after treatment with linsitinib no animal with fibrosis was detected (**Figures 7A, B**). None of these values were significant, if compared to controls.

### 3.6 Linsitinib improves inflammation, brown fat development and edema in TED

**Figure 8:**
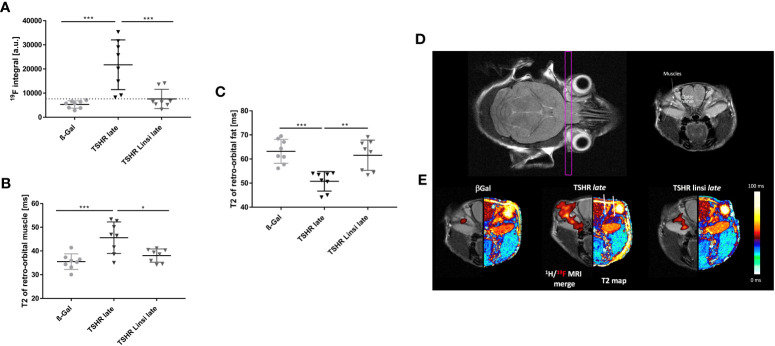
Effects of linsitinib on development of key features in the pathogenesis of TED, visualized in MR images of living mice. MRI was performed right before the sacrifice of the *late* TSHR immunized group, the *late* TSHR immunized + linsitinib treated group and the healthy control ß-Gal group. 8 mice per group were randomly selected for measurement. **(A)** ^19^F integral of PFC-loaded macrophages/monocytes are expressed in arbitrary units. (a.u.) **(B)** T2-weighted ^1^H MRI of the retro-orbital muscle is given in ms. **(C)** T2-weighted ^1^H MRI of the retro-orbital fat is given in ms. **(D)** Representative images of MR imaging of the mice. The images on the upper row, help to clarify the anatomical orientation. On the upper left side, the intersecting plane in which the images were created is visualized, whereas on the upper right side an image of the anatomical surroundings, including the optical nerve and the orbital muscles can be seen. **(E)** Representative images of ^19^F and T2 MR imaging of the mice. On the left side a 1H/^19^F merge image is displayed. On the right side a T2 map visualizes the effects in retro-orbital fat and muscle tissue. Arrows in the middle picture indicate to the surrounding fat (arrow on the left-hand side) and muscle tissue (arrow on the right-hand side). Presented are the mean ± SD **(A–C)** and representative images **(D, E)**, n=8 for ß-Gal, n=10 for TSHR *late* and TSHR Linsi *late*. Statistical differences were determined using one-way-ANOVA; *p < 0.05, **p < 0.001, ***p < 0.001.

To evaluate the effect of linsitinib during the course of therapy, living mice were examined via MRI. For MR imaging 8 randomly selected mice of the *late* TSHR-immunized group, *late* TSHR-immunized group treated with linsitinib and the healthy ß-Gal control group were selected. For direct monitoring of orbital inflammation, the animals were injected with Perfluorocarbon Nanoemulsion (PFC), which is greatly taken up by circulating monocytes/macrophages and can be detected via ^19^F MRI (27). ^19^F integral of PFC-loaded macrophages/monocytes are expressed in arbitrary units (a.u.) (**Figure 8A**). A marked accumulation of ^19^F signal, and thus distinctive migration of immune cells in the orbit with consecutive inflammation, was observed in the TSHR-immunized group (**Figure 8A**). This effect, and therefore the inflammation process, is completely blocked by treatment with linsitinib (**Figure 8A**). Accompanied with an increased ^19^F signal were the findings in the corresponding T2-weighted ^1^H MRI, in which the orbital inflammation was partnered with an T2 increase in the in the retro-orbital muscle in the TSHR-immunized group, indicating the development of edema (**Figure 8B**). This Increase in T2 and thus the onset of edema is greatly inhibited by treatment with linsitinib (**Figure 8B**). In order to determine the development of brown adipose fat tissue (BAT) in the orbit, again T2-weighted ^1^H MRI images were created. It has been shown, that BAT has substantially lower baseline T2 values than white adipose tissue (WAT) due to a higher amount of mitochondria in the BAT, higher blood flow and more metabolic activity (28). As seen in **Figure 8C**, the TSHR-immunized mice show significantly lower levels of T2 in the retro-orbital fat tissue, indicating a tissue remodeling of WAT to BAT. The browning and the subsequent development of BAT is blocked upon treatment with linsitinib (**Figure 8C**).

To visualize the effects, anatomically corresponding coronal 1H/^19^F merged MR images and a color coded T2 map were created (**Figure 8E**). The intersecting plane in which the images were created and the anatomical surroundings are shown as well (**Figure 8D**). The ß-Gal mice show a minimal baseline signal in the 1H/^19^F MRI merge (**Figure 8E**). This signal is markedly increased in the TSHR-immunized mice, whereas treatment with linsitinib greatly decreased the ^19^F signal, and thus inhibited the inflammation process (**Figure 8E**). The T2 map visualizes the effects of linsitinib in retro-orbital fat and muscle tissue (**Figure 8E**). In the TSHR-immunized group the fat tissue appears dark-red, which indicates a decrease of T2 in the fat tissue (see adjacent color chart) and thus a remodeling of WAT towards BAT (**Figure 8E**). On the other hand, the surrounding muscles in the TSHR-immunized group appears dark-blue, which indicates an increase of T2 in muscle tissue (see adjacent color chart) and thus development of edema (**Figure 8E**). As visualized in the T2 maps, linsitinib reversed these effects and thus inhibits the onset of edema, BAT and inflammation (seen in the 1H/^19^F merge) and therefore three key features in the pathogenesis of TED.

### 3.7 Linsitinib improves autoimmune hyperthyroidism, TED and the outcome of total Graves’ disease

**Figure 9:**
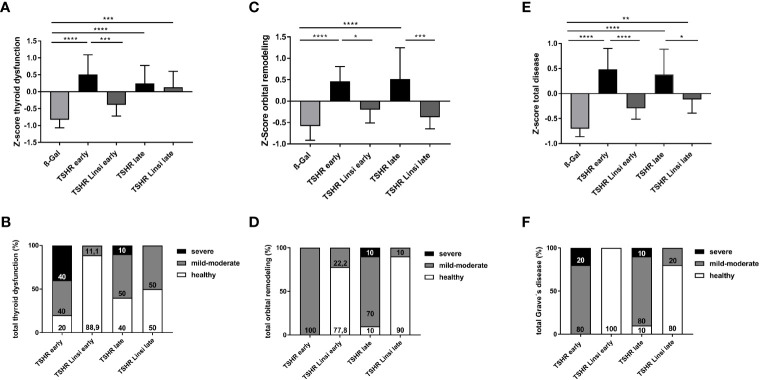
Total disease outcome of untreated and linsitinib treated, immunized mice. Results of different experiments were combined, normalized and analyzed using the Z-score method. The Z-score represents the value of standard deviation from the mean value of the total mouse population and is given in arbitrary units. **(A)** The Z-score for the thyroid dysfunction includes the stimulating activity of anti-TSHR autoantibodies (**Figure 2**), total anti-TSHR binding antibody titers (**Figure 2**), T4 values (**Figure 3**), thyroidal CD3+ T-cell infiltration (**Figure 3**) and weight change (**Figure 4**). **(B)** Disease classification was based on Z-score values. The number of mice is given in %. Subclinical disease (Z-score <0): These mice did not significantly manifest autoimmune hyperthyroidism and/or TED although these TSHR A-subunit immunized mice might have developed TSHR antibodies. Clinical disease (Z-score >0): These mice manifested a clinical disease during the experiment. Clinical disease is classified in accordance with Z-score values as mild-moderate (Z-score 0>Z<1) or severe (Z-score >1). The number of mice is given in %. **(C)** The Z-score for the orbital remodeling includes the orbital CD3+ T-cell infiltration in the orbit (**Figure 5**), brown adipose tissue enlargement (**Figure 5**), orbital macrophage infiltration (**Figure 6**) and fibrosis (**Figure 7**). **(D)** Disease classification was based on Z-score values as above. **(E)** The Z-score for the total disease summarizes the Z-score for the thyroid dysfunction and the Z-score for the orbital remodeling and represents the disease as a whole. **(F)** Disease classification was based on Z-score values as above. Presented are the mean ± SD **(A, C, E)** and disease classifications in percent **(B, D, F)**, n=8 for ß-Gal, n=10 for TSHR *early*, TSHR *late* and TSHR Linsi *late* and n=9 for TSHR Linsi *early*. Statistical differences were determined using one-way-ANOVA; *p < 0.05, **p < 0.001, ***p < 0.001, ****p < 0.0001.

In order to compare the disease outcomes between the different mouse groups, we used a Z-score method, as described earlier (20, 29). Here we combined, normalized and summarized the different parameters for each mouse to create a disease score for either thyroid dysfunction or orbital remodeling. The Z-score for the total disease outcome summarizes the two separate scores into one, reflecting the severity of the disease as a whole. For evaluation of the thyroid dysfunction 5 data sets were included in the Z-score: Titers of anti-TSHR stimulating autoantibodies (**Figure 2**), total anti-TSHR binding antibody titers (**Figure 2**), T4 values (**Figure 3**), thyroidal CD3+ T-cell infiltration (**Figure 3**) and weight change (**Figure 4**). As shown in **Figure 9A**, both the *early* and *late* TSHR-immunized group developed a severe thyroid dysfunction compared to the healthy ß-Gal group (**Figure 9A**). Treatment with linsitinib in the *early* TSHR-immunized group prevented the development of autoimmune hyperthyroidism (**Figure 9A**). Linsitinib had no significant effect on the thyroid dysfunction in the *late* group (**Figure 9A**).

In addition, the individual Z-scores of the mice were classified into different categories, analogous to the disease classification of TED severity in patients (30). Z-scores <0 indicate a healthy/subclinical mouse. Z-scores 0<Z<1 indicate a mild-moderate disease and a Z-score >1 represents a severe disease. In the *early* TSHR-immunized group 80% developed an autoimmune hyperthyroidism, with 40% having a mild-moderate thyroid dysfunction and 40% a severe thyroid dysfunction (**Figure 9B**), while 20% of the *early* TSHR- control group remained a normal/subclinical thyroid function (**Figure 9B**). Treatment of the *early* TSHR-immunized mice with linsitinib improved the disease and only 11.1% of the mice showed a mild-moderate autoimmune hyperthyroidism, whereas 88.9% showed a normal thyroid function (**Figure 9B**). In the *late* TSHR-immunized group, 50% developed a mild-moderate autoimmune hyperthyroidism, 10% a severe hyperthyroidism, and 40% of the animals had a normal thyroid morphology (**Figure 9B**). Linsitinib improved the *late* group and no severe cases were detected anymore after linsitinib. 50% of mice showed a mild-moderate thyroid dysfunction and 50% of the mice exhibited normal thyroids (**Figure 9B**).

For evaluation of the orbital dysfunction, 4 data sets were included in the Z-score: CD3+ T-cell infiltration in the orbit (**Figure 5**), brown adipose tissue enlargement (**Figure 5**), orbital macrophage infiltration (**Figure 6**) and fibrosis (**Figure 7**). Both the *early* and *late* TSHR-immunized developed severe thyroid eye disease compared to healthy ß-Gal mice (**Figure 9C**). Linsitinib improved outcome of the orbital remodeling in both, the *early* and *late* TSHR-immunized group (**Figure 9C**). Linsitinib had a stronger effect in the *late* treated group than in the *early* treated group. The beneficial effect of linsitinib on the disease is also indicated by the observation that both linsitinib-treated TSHR-immunized groups did not statistically differ from the healthy ß-Gal group (**Figure 9C**). The clinical classification shows that 100% of the *early* TSHR-immunized group developed a mild-moderate TED (**Figure 9D**). Linsitinib significantly improved this orbital remodeling and 77.8% of the mice were scored healthy, and only 22.2% mice were scored with mild-moderate TED, (**Figure 9D**). In the *late* TSHR-immunized group, 10% had a severe TED, 80% developed a mild-moderate TED and 10% were healthy (**Figure 9D**). Linsitinib almost completely normalized the orbital dysfunction in the *late* group, with only 10% having a mild-moderate TED, 90% of the mice were scored healthy and none developed a severe TED (**Figure 9D**).

To provide an overview of the total disease score, we summarized the two individual scores for thyroid dysfunction and orbital remodeling into one total disease score (**Figure 9E**). The *early* and *late* TSHR-immunized group developed severe GD compared to the healthy ß-Gal group (**Figure 9E**). In the *early* group linsitinib had a highly significant effect on GD development (p ≤ 0.0001) (**Figure 9E**). The outcome of the total GD also significantly improved in the *late* group (p ≤ 0.05) (**Figure 9E**). In the *early* TSHR-immunized group, 20% of the mice developed a severe disease and 80% a mild-moderate GD (**Figure 9F**). Treatment with linsitinib in the *early* group resulted in 100% healthy mice and thus completely blocked the development and progression of GD (**Figure 9F**). In the *late* TSHR-immunized group, 10% of the mice developed a severe GD, 80% a mild-moderate disease, and 10% remained subclinical (**Figure 9F**). Treatment with linsitinib in the *late* group resulted in 80% healthy mice and only 20% with mild-moderate cases (**Figure 9F**).

## 4 Discussion

In the present study, we examined the effects of linsitinib, an orally available, a dual small-molecule kinase inhibitor of the insulin-like growth factor 1 receptor (IGF-1R) and the Insulin receptor (IR) on disease progression in an experimental murine model of GD. We demonstrate that linsitinib effectively improves the total disease outcome in animals immunized with a hTSHR A‐subunit. Application of linsitinib in the *early* state of the disease completely prevented the development of autoimmune hyperthyroidism. If linsitinib was applied during disease development, i.e., in a more chronic state of the disease, linsitinib has its main effect on orbital remodeling, which the drug effectively inhibited. The drug prevented further progression of experimental GD. In addition, we show that treatment of immunized mice with linsitinib after disease onset significantly limited the severity of the disease.

### 4.1 Reproducibility of our mouse model

Subsequent studies showed the model to be robust in variable environmental exposure and to reflect characteristic and stable hallmarks of TED pathology comparable to the situation in patients (29, 31). In multiple studies using this mouse model we have investigated the natural course of the disease (32) elucidated sex differences during the TED development (22), determined the role of the microbiome on disease outcome (33) and examined the efficacy of potential therapeutic treatment options (20).

### 4.2 Crosstalk between the TSHR and the IGF-1R

Krieger et al. (34) previously showed that treatment of Graves’ orbital fibroblasts with M22, a high affinity human monoclonal stimulating TSHR antibody (35), resulted in a synergistic stimulation of hyaluronan acid secretion, which plays a pivotal role in the pathogenesis of TED. In addition, Tsui et al. (9) displayed evidence for crosstalk between the TSHR and IGF-1R in thyroid tissue. Thyrocytes treated with recombinant human TSH showed a rapid increase of phosphorylated ERK, which was completely blocked when combined with 1H7, an anti-IGF-1R blocking monoclonal antibody, which acts similar to linsitinib (9). These findings support our data that linsitinib prevents autoimmune hyperthyroidism in the *early* state of the disease.

### 4.3 Effect of linsitinib on immune cells

In addition, linsitinib might have a direct effect on T-lymphocytes. T-lymphocytes, including Th17 and Tregs, are very important in autoimmune disorders and control immune activation (36). In particular, patients with severe TED show a significant increase in TH17-cells in the serum (37). Th17-cells mediate pro-inflammatory responses, whereas Treg cells play an important role in modulating the autoimmune system and tolerance to self-antigens and a well-regulated Th17/Treg balance is crucial to determine the function of the immune system (36).

CD4+ T-cells are known to express IGF-1R and are targets of IGF (38). Moreover, Douglas et al. (5) detected an abnormal high level of T-cells expressing the IGF-1R in blood samples of patients suffering from GD and TED. DiToro et al. (39) have recently shown an alteration of the Th17/Treg balance, in which IGF-signaling through activation of the AKT-mTOR pathway promotes the differentiation of Th17 cells and on the other hand suppresses the development of Treg cells, resulting in a pro inflammatory immune response. For TED, it was shown in a recently published paper, that blocking the Akt/mTOR pathway, and thus an important downstream pathway for IGF-signaling, with Sirolimus, achieved a significantly better anti-inflammatory treatment effect compared to first line therapies, such as intravenous methylprednisolone, in the treatment of active TED (40).

Thus, it is very well possible that linsitinib prevents the TCR/CD3-mediated activation of T-lymphocytes to the TSHR antigen and thereby reduces T-lymphocyte numbers in the orbita and inflammation. Further, linsitinib might promote the differentiation and/or activation of Tregs at least in the setup of autoimmune disorders and thereby also prevent the development of the autoimmune disease. By blocking the IGF-1R mediated signaling through the AKT-mTOR pathway, linsitinib might have a reverse effect on the Th17/Treg balance, by suppressing the proinflammatory cytokine Th17 and enhancing Treg development and therefore has a positive effect on the outcome of Graves’ disease. If linsitinib is definitively shown to impact impacts the Th17/Treg balance, then one can conclude, that clinical practice may include treatment with linsitinib as early as possible upon diagnosis in order to primarily prevent a severe course of TED. However, IGF-1R has also shown to inhibit inflammatory responses (41) and the ultimate function of IGF in regulation of anti-inflammatory vs. pro-inflammatory responses seems to be dependent on stimulation context and co-stimuli (42).

We noted that some mice in the *late* group responded to the linsitinib treatment with a decrease of TNFa and thus decrease of infiltrating, proinflammatory M1-macrophages, while a few mice did not respond to the linsitinib treatment with a lower number of infiltrating macrophages. Since linsitinib had no effect on macrophage infiltration in the *early* group, it might be possible that the drug requires more time to reveal its effect on macrophage infiltration. Thus the lack of an effect of linsitinib on macrophage infiltration in the *early* group could be explained that a longer treatment is required to detect an effect on the macrophages. In the *late* group we might be at a “break-point”- for some mice, in which we are at the right time and decreased the macrophage infiltration in one group of mice, however for some mice the timing was too early. Given the immunization processes, with a variable uptake of the TSHR A-subunit plasmid, this could explain why some mice already responded to the treatment and why some mice might respond to a later timepoint.

### 4.4 Role of brown adipose tissue and the effect of linsitinib

One of the main problems in TED is the dysregulation of the orbital adipose tissue (OAT).

Orbital adipose tissue differs from other locations in the body. There is sufficient evidence that the unique adipocyte biology of OAT facilitates expansion of OAT in TED. It has been shown from several groups, that the direct stimulation of the TSHR in the orbit leads to a differentiation of orbital preadipocytes to mature adipocytes (43–46). In contrast to white adipose tissue (WAT), brown adipose tissue (BAT) is involved in adaptive thermogenesis and is derived from Myf5+ mesenchymal stem cells (MSCs), whereas WAT is derived from Myf5- MSC (47). In addition, the presence of BRITE (BRown in whITE) adipose tissue in adults has been identified, also derived from Myf5- MSCs which can convert into WAT or BAT depending on the circumstances (48). Orbital fibroblasts, possess mesenchymal stem cell features, by for example expressing CD90, which is a MSC marker (49) and can differentiate into adipocytes under the appropriate circumstances. In TED, activation of the TSHR in orbital fibroblasts leads to an increased adipogenesis as well as an increased hyaluronan production (50) In addition, hyperthyroidism is known to induce brown fat activity in BAT and BRITE fat (51). Ex vivo analysis of fat samples from TSAB positive GD patients revealed a significant increase of WAT and BRITE transcript markers in comparison to TSAB negative controls (52, 53). Previosuly, the importance of the TSHR and IGF1R crosstalk has been reported, which plays an essential role in the OAT expansion in TED via the IGF1R/PI2K/FOXO signalling pathway (54). In addition, the FGF signalling pathway has been shown to play an important role in OAT expansion of TED patients, which leads to an activation of IGF2 which binds to the IGF-1R, leading to an activation of the IGF2/IGF1R axis (54, 55). This goes in line with our findings that linsitinib inhibits the formation of BAT in mice suffering from TED and thus prevents the ongoing remodling in the orbita by blocking the IGF-1R and TSHR crosstalk.

### 4.5 Role of sphingolipids in autoimmune hyperthyroidism

Previous studies from our group demonstrated an important role of sphingolipids, in particular sphingosine 1-phosphate in remodeling of the orbital tissue upon induction of TED (56–58). This was mediated by an upregulation and activation of CD40 in orbital fibroblasts (56). It will be very interesting to investigate whether linsitinib also has an influence on this pathway and whether linsitinib regulates the local effects of sphingosine 1-phosphate. The present study describes the animal and the disease model to perform these studies and describes a framework to define the function of sphingolipids in thyroid eye disease.

### 4.6 Linsitinib prohibits autoimmune hyperthyroidism

Further, our studies indicate that treatment with linsitinib normalizes autoimmune hyperthyroidism. Several groups have shown in *in vivo* and *in vitro* models that the TSH/IGF-1R cross-talk also plays an important role in the regulation of the thyroid function (7, 59–63).

Morgen et al. (2016) showed that TSHR/IGF-1R crosstalk upregulates the Sodium-Iodide symporter expression in primary cultures of human thyrocytes, which is essential for thyroid hormone production. Linsitinib inhibited the upregulation of the Sodium-Iodide symporter in TSH-stimulated cells (61). Therefore, it can be concluded, that linsitinib can also inhibit the action of stimulating anti-TSHR antibodies. Our data strongly support such a role of IGF-1R and suggest that stimulation of the TSH receptor by the autoantibodies requires IGF-1R or is at least facilitated by IGF-1R. In this scenario hyperthyroidism is induced by the autoantibodies that bind to TSH-receptor, but activation of the receptor-dependent signaling machinery and thereby stimulation and proliferation of thyrocytes is mediated by the coordinated interplay of TSHR and IGF-1R, which is blocked by linsitinib.

However, in contrast to the clearly visible hyperthyroidism found in the histopathology, not all mice suffering from hyperthyroidism show elevated T4 levels. This is a topic, which our group is currently working on.

### 4.7 Euthyroid thyroid eye disease

Most of the changes in the orbita are consistent with effects expected in the context of hyperthyroidism. However, some mice only exhibited orbital remodeling. Two mice in the early TSHR immunized group did not show an autoimmune hyperthyroidism but developed moderate TED symptoms. In the late TSHR immunized group 4 mice did not develop an autoimmune hyperthyroidism or the thyroid dysfunction was declining to euthyroid conditions. However, all of those mice developed mild-moderate TED symptoms. Of those mice 3/6 revealed stimulating anti-TSHR antibodies above the 95% of the control ß-Gal mice, whereas one mouse in the early TSHR immunized group and two mice in the late TSHR immunized group did not develop stimulating anti-TSHR antibodies.

In humans it has been shown that TED can develop in euthyroid and even in hypothyroid patients but almost all of the patients have in common, that they show positive stimulating anti-TSHR antibody levels, although at a much lower degree than hyperthyroid patients. Even in the euthyroid/hypothyroid patients the levels of the stimulating anti-TSHR antibodies correlated with severity of TED (64).

### 4.8 Development of fibrosis in TED

A recent study by Zhang et al. (65) demonstrated fibrosis in a mouse model for TED. In their studies TED was induced by 9 immunizations using an adenovirus system expressing the TSHR-A subunit. Fibrosis did only develop after 9 immunizations, but was absent after 4 or 7 immunizations (65). Our studies employed only 3 immunizations and in accordance with the studies by Zhang et al. (65) we did not detect a significant fibrosis during this shorter immunization protocol and experimental time course, which may not drive a persistent autoimmune reaction. However, we detected a trend to development of fibrosis, which was evident in 3 out of 10 mice of the *late* TSHR immunized group and importantly not in the *late* TSHR immunized mice which were treated with linsitinib, indicating a suppression of fibrosis development in the *late* state of the disease. Future experiments with a higher number of immunizations and longer observation periods will elucidate the development of fibrosis in our TED model.

### 4.9 Significance of our findings and comparison to other IGF-1R inhibitors

Other IGF-1R inhibitors are used for therapy, such as the fully humanized monoclonal antibody teprotumumab that inhibits activation of IGF-1 receptor by blocking autophosphorylation and inducing degradation and internalization of the IGF-1R via b-arrestin-1 activation. In the past few several years clinical studies have been performed showing an improvement of the disease in 70% of TED patients upon treatment with teprotumumab, an intravenously administered antibody. However, these promising results are accompanied with severe side effects like hearing loss (in 7% of the patients), nausea (20% of the patients) and muscle spasms (20% of the patients) (66–69), accompanied with a relapse rate of around 40% (70). In addition, teprotumumab is currently only approved for TED treatment in the United States. Therefore, substantial need exists in the population of people with TED for additional safe and effective treatment options.

For linsitinib, an overall good safety profile has been reported in several cancer studies (11, 14), with gastrointestinal side effects (nausea, constipation, vomiting) and fatigue being the most commonly observed side effects. Hyperglycemia has been reported as a side effect as well (11), whereas other clinical trials did not report hyperglycemia as a side effect (14, 71). In a study with cancer patients suffering from diabetes and treated with linsitinib, hyperglycemia was more common than in the non-diabetic groups, however no alteration in diabetes medications was required during the study and no patients had clinically significant elevated glycosylated hemoglobin or lactate levels (11). For this reason, the drug was evaluated as being safe for diabetic patients as well (11).

## 5 Conclusion

In summary, we demonstrate the development of GD and TED in a mouse model upon immunization against the TSHR. The oral IGF-1R inhibitor linsitinib blocks the development of the local pathologies of GD and TED in an *early* and *late* phase of the autoimmune disorder and also prevents development of the autoimmune response. We show that treatment of immunized mice with linsitinib after disease onset significantly limited the severity of the disease, indicating the clinical significance of the findings and providing a path to therapeutic intervention of Graves’ Disease and TED.

## Data availability statement

The original contributions presented in the study are included in the article/supplementary material. Further inquiries can be directed to the corresponding author.

## Ethics statement

The animal study was reviewed and approved by North Rhine Westphalian State Agency for Nature, Environment and Consumer Protection (LANUV), Germany.

## Author contributions

AG: Conceptualization, Methodology, Validation, Formal analysis, Investigation, Writing–Original draft preparation, Visualization, Project administration. MH: Formal analysis, Investigation. AD: Expertise. UF: Resources, Methodology. MO: Resources. NB: Resources. JB: Expertise, Supervision. SK: Formal analysis. BW: Formal analysis. GK: Expertise, Writing- Reviewing and Editing. GH: Funding source. AS: Funding source, Supervision. RZ: Funding source, Supervision. AE: Resources, supervision, Writing- Reviewing and Editing, Project administration, Funding acquisition. SP: Conceptualization, Methodology, Resources, supervision. G-EG Conceptualization, Methodology, Resources, supervision, Writing- Reviewing and Editing, Project administration. All authors contributed to the article and approved the submitted version.
